# Gastrointestinal Symptoms After Sport-Related Concussion in Irish Athletes

**DOI:** 10.3390/nu18060914

**Published:** 2026-03-13

**Authors:** Emma Finnegan, Ed Daly, Katherine J. Hunzinger, Lisa Ryan

**Affiliations:** 1 Department of Sport, Exercise and Nutrition, School of Health, Sport Science and Nutrition, Atlantic Technological University (ATU), Dublin Rd., H91 T8NW Galway, Ireland; emma.finnegan@research.atu.ie (E.F.); ed.daly@atu.ie (E.D.); 2 Department of Exercise Science, Thomas Jefferson University, Philadelphia, PA 19144, USA; katherine.hunzinger@jefferson.edu; 3 Jefferson Center for Injury Research & Prevention, Thomas Jefferson University, Philadelphia, PA 19144, USA

**Keywords:** mild traumatic brain injury, concussion, gastrointestinal symptoms, gut–brain axis, sport injury, athlete recovery, nutrition, digestive health, energy

## Abstract

**Background/Objectives**: Sport-related concussion (SRC) elicits multi-systemic symptoms, including nausea, fatigue, and cognitive changes. Gastrointestinal (GI) symptoms are not well captured in current concussion assessments and may be under-recognised in clinical follow-up. GI disturbances may influence intake tolerance and day-to-day fuelling during post-SRC recovery. This study investigated the prevalence and severity of self-reported GI symptoms in Irish athletes after their most recent SRC, examined sex-based patterns, and evaluated the rationale for integrating GI symptom checks into standard concussion tools (e.g., SCAT6) and post-injury monitoring. **Methods**: An online survey was completed by recreational, competitive, and elite athletes who retrospectively self-reported concussion history, GI symptoms, and bowel function post-SRC and at the time of survey completion (ToSC; 0.03–216 months post-injury). The survey used the Bristol Stool Chart, Rivermead Post-Concussion Symptoms Questionnaire, and validated GI symptom measures. Descriptive statistics and chi-square tests examined timepoint- and sex-based differences. **Results**: A total of 106 athletes participated (55.7% female; mean age 26.4 ± 7.7 years), of whom 90.6% reported ≥1 GI symptom post-SRC, with greater severity observed for appetite loss, bloating, and abdominal discomfort. Bowel habits shifted bidirectionally for 42.5%, and 26.4% were experiencing ongoing symptoms at ToSC. **Conclusions**: Self-reported GI symptoms were common and appear under-recognised post-SRC. These findings support greater attention to GI symptom assessment and suggest that brief GI checks and facilitated access to nutrition advice where symptoms persist may be feasible within multidisciplinary, athlete-centred care. Prospective studies are needed to determine clinical relevance and to evaluate nutrition-related strategies.

## 1. Introduction

Sport-related concussion (SRC) is a form of mild traumatic brain injury (mTBI) caused by direct or indirect forces to the head or body, resulting in rapid brain movement and initiating neurometabolic, autonomic and neuroinflammatory disturbances [1,2,3]. In Ireland, collision (e.g., rugby), contact (e.g., Gaelic football, hurling, camogie), and combat sports (e.g., boxing) involve frequent high-impact events, posing a recognised risk of SRCs and repetitive head impacts (RHIs) [4,5].

Despite growing awareness, SRC remains challenging to recognise due to heterogeneous symptom presentations, the absence of objective biomarkers, and reliance on athletes to self-report [1,6,7,8]. Under-reporting is common and influenced by internal factors such as fear of lost playing time, and external pressures, making accurate incidence estimation and injury documentation difficult [6,7,9,10,11], contributing to incomplete injury surveillance [12] and persistent global under-reporting [13].

SRC symptoms span somatic, cognitive, emotional, sleep, and autonomic nervous system (ANS) domains [14,15]. Emerging evidence suggests that gastrointestinal (GI) symptoms such as nausea, bloating, and altered bowel habits may also occur following SRC [15,16]. These symptoms are clinically relevant because GI disturbances can affect fuelling, hydration, and tolerance to rehabilitation during a period of increased metabolic demand [4], potentially contributing to greater symptom burden. However, current concussion assessment frameworks, including the Concussion in Sport Group (CISG) guidelines and the Sport Concussion Assessment Tool, 6th Edition (SCAT6) [1,7], prioritise neurological and cognitive symptoms and capture minimal GI-related information [17,18].

Athlete-reported data indicate broader post-concussive GI disturbances, with potential variation by sex and recovery stage [19,20,21]. Biological sex differences, including hormonal influences, may contribute to variability in GI symptom presentation, prevalence, and severity. However, these dimensions are rarely incorporated into existing concussion assessment tools, which remain insensitive to GI-related or sex-specific symptom profiles [19,20,21].

The gut–brain axis (GBA), a bidirectional communication network linking the central (CNS) and enteric (ENS) nervous systems, immune pathways, and neuroendocrine signalling, may contribute to post-concussive symptom patterns [15,21,22]. The gut microbiome plays important roles in digestion, immune regulation, appetite control, nutrient absorption, hormonal balance, and metabolic homeostasis, functions that may be altered following SRC [4,21,23,24,25,26,27]. Disruptions to gut–brain communication may influence inflammation, metabolic regulation, energy availability, and neural repair, potentially prolonging symptoms, although evidence in SRC remains limited [4,21,25]. Biological sex hormones (e.g., oestrogen, progesterone) may further modulate gut motility and microbiome composition, potentially contributing to sex-specific GI symptom profiles [6,10,15,24,25]. Despite these biologically credible pathways, GBA dysfunction remains underexplored in SRC compared to other neurological conditions such as depression, Alzheimer’s disease, Parkinson’s disease, and stroke [28,29].

GI or GBA disturbances following SRC may affect appetite or tolerance to food intake, which can make fuelling more difficult during periods of elevated metabolic demand. Although plausible, effects on digestion or nutrient absorption have not been established in populations post-SRC. Reduced digestion, uptake, and energy availability can limit access to nutrients [4] including protein and omega-3 fatty acids that may support brain recovery and neural repair [20]. No standardised nutritional guidelines exist for athletes recovering from SRC or mTBI, and current recommendations include limited guidance beyond the acute phase [4]. This gap underscores the need for integrated assessment models that consider GI and GBA-related disturbances not captured by existing tools such as SCAT6.

This study aimed to characterise self-reported GI symptoms among Irish athletes following their most recent SRC and to explore the potential clinical relevance of incorporating GI symptom checks into concussion assessment frameworks. We hypothesised that (i) GI symptoms would be prevalent following SRC (defined as >50% of participants reporting ≥1 GI symptom post-SRC), and (ii) sex-based differences in symptom prevalence and characteristics would be observed. Time since the most recent concussion/mTBI was included to contextualise symptom reporting.

## 2. Materials and Methods

This Irish study obtained ethical approval from the Atlantic Technological University’s Ethics Board (ATU-RSC_AC_15/12/2023) on 15 December 2023 and was conducted in accordance with the Declaration of Helsinki (2013). A cross-sectional survey design was used to examine the prevalence and characteristics of self-reported GI symptoms post-SRC among Irish athletes and explore their potential clinical relevance.

### 2.1. Participants and Study Design

Eligible participants were athletes with a self-reported history of concussion or mTBI. For this study, concussion/mTBI events occurring during sport were treated as SRCs. The survey comprised 49 items (available upon request) and required full completion prior to submission. It collected data on demographics, injury history, sporting background (past and present), GI health and function, and post-concussion symptomatology. Items referred to participants’ status at the time of survey completion (ToSC) or their experiences immediately following their most recent SRC. Participants also reported the time elapsed since this event, which was used to characterise the sample and to anchor all post-injury symptom reporting to the most recent SRC; no time limit was imposed. No comparisons across time-since-injury subgroups were conducted.

Participants self-reported the total number of concussions or mTBIs they had sustained and indicated how many were medically diagnosed (e.g., by a doctor, physiotherapist) or self-diagnosed. A standardised definition of concussion and mTBI was provided, and response options ranged from 1 to 9 and 9+, to ensure consistency across responses (Supplementary Table S1). Full details of the assessments administered are provided in Supplementary Table S2 (GI-specific) and Table S3 (Rivermead Post-Concussion Symptoms Questionnaire; RPQ) [30].

#### 2.1.1. Sample Recruitment

Participants were recruited using convenience sampling via email, social media (Instagram, LinkedIn, X, Facebook), and poster distribution. The survey was shared with sporting organisations, university sports departments, national players’ associations, and club administrators using publicly available contact information, with additional targeted outreach to athletes and support staff (e.g., coaches, nutritionists, physiotherapists). Participation was voluntary and anonymous. Before beginning the survey, participants reviewed an information sheet detailing the study purpose and procedures and provided electronic informed consent. To maintain confidentiality, no personally identifiable information (e.g., names) was collected, and all responses were stored anonymously on encrypted, password-protected institutional cloud storage (OneDrive) accessible only to the research team. Data collection took place between January and August 2024 via Microsoft Forms (Version 2502; Microsoft Corporation, Redmond, WA, USA).

#### 2.1.2. Gastrointestinal Function Assessment

GI function was assessed using the Bristol Stool Form Scale (BSFS) for two conditions: (1) athletes’ typical or current function at ToSC, and (2) their experience immediately following SRC. Participants rated stool form according to the standardised BSFS categories, allowing comparison of pre- and post-injury GI function (Supplementary Table S4) [31].

#### 2.1.3. Post-Concussion Symptom Assessment (General and GI-Specific)

Post-concussion symptoms were assessed using the 16-item RPQ, a validated measure of somatic, emotional, and cognitive symptoms (Supplementary Table S3) [30,32]. Twenty GI-specific symptoms (Supplementary Table S2) were adapted from validated instruments, including the Functional Gastrointestinal Disorders (FGD) Symptom Questionnaire and the Gastrointestinal Symptom Rating Scale (GSRS) [33].

Symptom prevalence, frequency, and severity were rated retrospectively on a modified five-point Likert scale (0 = “not experienced” to 4 “severe problem”) for both the RPQ and GI items. Scores ≥1 indicated symptom presence, with mild-to-severe symptoms defined as ratings 2–4. A standardised definition of recovery was not provided to participants; therefore, symptoms reported at the retrospective 3-month, 6-month and ToSC ratings were classified as ongoing. Participants reported their GI function at three months and six months post-SRC and at the ToSC. Response categories: (1) poor gut health; (2) manageable GI symptoms; (3) normal or good GI function; or (4) no symptoms. Additional items captured dietary changes, supplement use, and access to professional care. Symptom timepoints reflect post-injury experiences and current status at ToSC.

### 2.2. Statistical Analysis

Data cleaning and analysis were conducted using Microsoft Excel (Version 2502; Microsoft Corporation, Redmond, WA, USA). Missing data were minimal (0.02% of all responses; 1/5194) and occurred for one item (Q17); this was managed using available-case (pairwise) analysis (*n* = 105), with no imputation applied.

Descriptive statistics (frequencies, percentages, means, standard deviations [SD], medians, ranges, and 95% confidence intervals [CIs]) were used to summarise demographic characteristics, concussion history, and GI symptom prevalence and severity. Responses of “9+” concussions (*n* = 4) were top-coded as 10 to enable quantitative analysis. Sports were categorised by contact level (high; moderate; minimal/noncontact) and by mode of play (player-based team; stick-based team; combat; extreme; individual) (Table 1) [34]. Competitive level was self-reported as recreational, competitive, or elite. Statistical significance was set at *p* < 0.05. Effect sizes were calculated using Cramér’s V for chi-square tests (*χ*^2^), rank-biserial correlations for Mann–Whitney *U* tests, and absolute risk differences (*ARD*), odds ratios (*OR*), and relative risks (*RR*) for prevalence comparisons.

Statistical assumptions were tested; parametric tests were used when met, and non-parametric when not. Independent-samples *t*-tests were used for normally distributed variables (e.g., age), while Mann–Whitney U tests were used for non-normal continuous or ordinal variables (e.g., SRC counts). Symptom prevalence comparisons employed chi-square (*χ*^2^) tests or Fisher’s exact tests when expected cell counts were <5.

Symptom severity scores (ordinal ratings 1–4) were analysed only among participants reporting ≥1 symptom. Symptom burden was calculated as the total and mean number of symptoms reported at any severity (ratings 1–4) and mild-to-severe levels (ratings 2–4). Analyses included those with GI symptoms (*n* = 96 of 106) and RPQ symptoms (*n* = 105 of 106). A Symptom Severity Index (SSI) was used to rank the most impactful GI symptoms.

Paired stool-related variables (e.g., Bristol Stool Scale post-SRC vs. ToSC) were assessed using Wilcoxon signed-rank tests. Current stool type, post-SRC stool type, and bowel movement frequencies were compared using Mann–Whitney U tests. Fisher’s exact test was applied when expected values were <5. Changes in bowel frequency (post-concussion to ToSC) were analysed using paired *t*-tests and Spearman’s rank-order correlation (*ρ*).

Self-reported GI function (poor; manageable; normal/good) at 3 months, 6 months, and ToSC was summarised descriptively. Free-text responses were analysed qualitatively to identify ongoing or improving symptoms. Spearman’s rank-order correlations examined associations between gut/GI function, total GI symptom burden, and SRC counts.

## 3. Results

This study comprised 106 Irish athletes (59 females, 47 males; mean age 26.4 ± 7.6 years) who had experienced at least one SRC and completed the survey. All participants were included in analyses of GI symptom prevalence and characteristics (Table 1).

### 3.1. Concussion History and Diagnosis

All participants reported ≥1 concussion or mTBI, totalling 298 SRCs (mean 2.8 ± 2.3 per participant). Most SRCs were professionally diagnosed (65.4%, 195 cases reported by 51 participants) and occurred during sport (89.6%). Self-diagnosed SRCs accounted for 34.6% (103 cases, 89 participants), and 17 participants reported suspected SRC without a formal diagnosis. Among 105 participants (99.1%), the time since the most recent SRC ranged from <1 day to 216 months (mean 39.6 ± 44.3 months; median 24 months; IQR 6–60 months). Males reported significantly longer intervals than females (*t* (103) = −2.27, *p* = 0.026, *d* = −0.45).

### 3.2. Sport Participation and Contact Level

Most participants (84.9%, *n* = 90) reported involvement in at least one high-contact sport; among these, 52.2% were female and 47.8% male. Across all reported sports (n = 281) high-contact activities accounted for 69.0% (194 of 281) of all reported. Moderate-contact sports represented 16.4% (46 of 281), and minimal-to-non-contact sports 14.6% (41 of 281). Males were more likely to play high-contact sports (*p* = 0.027), while females were more represented in moderate-contact and minimal-contact sports (*p* < 0.001). Percentages reflect the distribution of reported activities (n = 281), as many participants engaged in multiple sports.

Player-based team sports were most common (57.3%, *n* = 161), followed by combat sports (16.0%, *n* = 45) and stick-based team sports (12.8%, *n* = 36). Most athletes competed at competitive (51.9%) or elite (44.3%) levels.

### 3.3. Gastrointestinal Function at ToSC and Post-SRC

#### 3.3.1. Bowel Movement Frequency

Reports of infrequent bowel movements (“once every 4–6 days”) increased from 1.9% at ToSC to 7.5% post-SRC (*p* = 0.052, *RR* = 4.50; Table 2). Reports of “2–3 times a day” also increased (*p* = 0.344, *RR* = 1.36), while “once a day” decreased (*p* = 0.490; *RR* = 0.90). Overall, mean bowel movement frequency declined significantly post-SRC (3.90 ± 1.13) compared with ToSC (4.19 ± 0.93, *t* (105) = −2.87, *p* = 0.005; Cohen’s *d* = 0.28).

Among participants reporting changes (46.2%, *n* = 49), 38 reported decreased frequency (constipation pattern), and 11 reported increased frequency (diarrhoea pattern). Most decreases were mild to moderate (71.4%; −1 point: *n* = 29; −2 points: *n* = 6), with larger decreases in 2.8% (−3 points). Changes ranged from “once daily” to “once weekly” (*n* = 1) and from “2–3 times daily” to “once every 4–6 days” (*n* = 2). Increases were mild in 22.4% (*n* = 11; +1 point), while four participants showed large increases (+3 to +4 points), moving from infrequent (“once a week” or “every 2–3 days”) to multiple daily evacuations.

Bowel movement frequency at ToSC and post-SRC showed a moderate positive correlation (Spearman’s *ρ* = 0.576, *p* < 0.001). A Mann–Whitney test indicated greater variability post-SRC (*U* = 8094.5, *Z* = 5.55, *p* < 0.001), demonstrating bidirectional changes.

#### 3.3.2. Stool Consistency

Normal stool forms (BSFS type 3–4) were most common at both timepoints (ToSC: 3.40 ± 0.95; post-SRC: 3.61 ± 1.42). Type 3 decreased for 49 participants (46.2%) at ToSC to 34 (32.1%) post-SRC (*p* = 0.027, *RR* = 0.69), and type 4 decreased for 36 participants (34.0%) at ToSC to 24 (22.6%) post-SRC (*RR* = 0.67). Softer stools (types 5–7) became more frequent post-SRC: type 5 increased from 2.8% to 14.2% (*p* = 0.001; *RR* = 5.00), and type 7 emerged in 2.3% of participants who had reported type 4 at ToSC (*p* = 0.054).

Overall, 52 participants (49.1%) reported stool-form changes, with 31 (29.2%) shifting toward softer stools (*RR* = 1.48) and 21 (19.8%) toward harder stools (*RR* = 1.68). Mean stool form scores increased slightly post-SRC (difference 0.22 ± 1.50); this change was not statistically significant (*t* (105) = 1.49, *p* = 0.139; Cohen’s *d* = 0.145). Stool form changes post-SRC correlated moderately with bowel movement frequency (Spearman’s *ρ* = 0.318).

#### 3.3.3. Bowel Frequency and Stool Consistency Associations

Bowel movement frequency was significantly associated with stool form. Higher frequency (≥2–3 times/day or more) was linked with softer stools (types 5–7; *p* < 0.001, *RR* = 5.00), whereas lower frequency (≤once every 2–3 days or less) was associated with harder stools (type 1–2; *n* = 8).

Following SRC, athletes shifted away from a once-a-day pattern (47.2%, *n* = 50 at ToSC) toward more variable bowel habits (42.5%, *n* = 45 post-SRC). Among those with higher frequency (≥2–3 times/day), soft stools increased from 5 participants (4.7%) at ToSC to 10 (9.4%) post-SRC (*p* < 0.001, *RR* = 3.20). Among those with lower frequency (≤once every 2–3 days), harder stools increased from 7 (6.6%) to 13 (12.3%; *p* = 0.023, *RR* = 0.31). Post-SRC, both softer stools (type 5–7, increased by 19 reports, 25.5%) and harder stools (type 1–2, increased by eight new reports, 19.9%) increased compared with ToSC. Overall, bowel habits displayed bidirectional changes, with both increases and decreases in frequency and stool consistency observed post-SRC.

#### 3.3.4. Self-Reported GI Function and Stool Patterns Post-SRC

Self-rated GI function post-SRC was generally moderate: 19 (17.9%) reported poor or fair, 41 (38.7%) moderately good, and 46 (43.4%) good or very good (median 3; IQR 3–4). Among those ratings of poor or fair GI function, 14 reported stool-pattern changes (six softer, eight harder). In the moderately good group, 14 reported softer stools post-SRC and 7 harder stools; in the good or very good groups, 11 reported softer stools and 6 harder.

Notably, reports of infrequent bowel movements (e.g., “once every 4–6 days”) increased (*p* = 0.052), alongside softer stools, particularly type 5 (*p* < 0.001) and type 7 (*p* = 0.054) post-SRC. Conversely, type 3 stools declined significantly (*p* = 0.021). Reported post-SRC changes included both increases in softer stools and increases in harder stools.

### 3.4. GI Symptom Presence Post-SRC

Out of 106 participants, 96 (90.6%) reported at least one GI symptom (ratings 1–4). Among these, 88 (91.7%, out of 96) experienced at least one symptom rated ≥2 (ratings 2–4), indicating worsening relative to baseline. Eight participants (8.3%) reported symptom severity 1 (present but no worse than usual), most commonly nausea (*n* = 6), appetite loss (*n* = 5), and tiredness (*n* = 4), with food cravings, abdominal pain, bloating, indigestion, and dry skin less frequent (*n* = 3).

**Figure 1:**
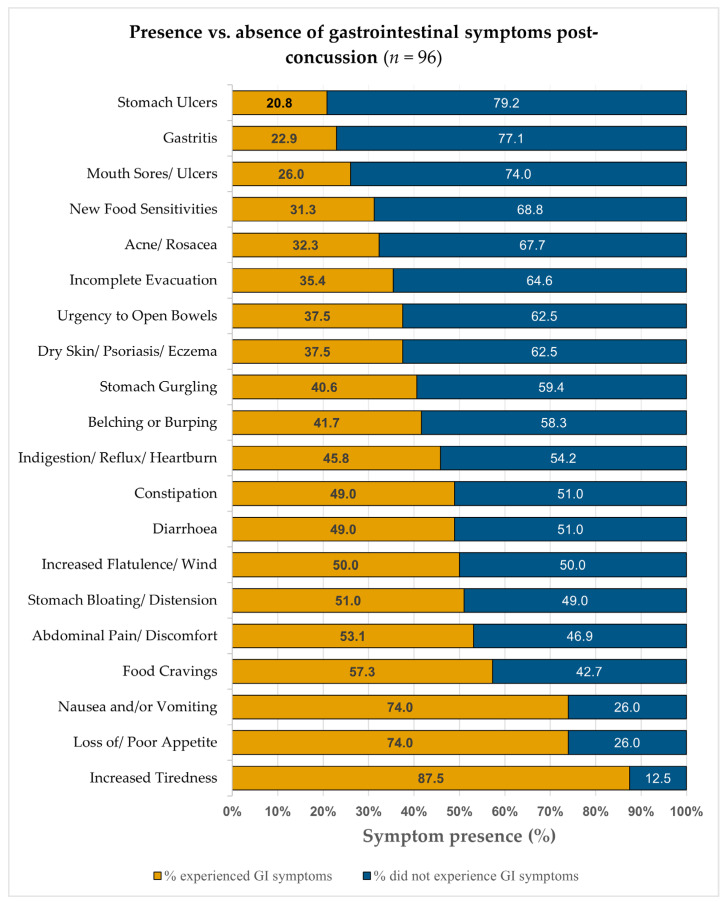
Presence versus absence of self-reported gastrointestinal (GI) symptoms post-concussion among participants.

**Figure 2:**
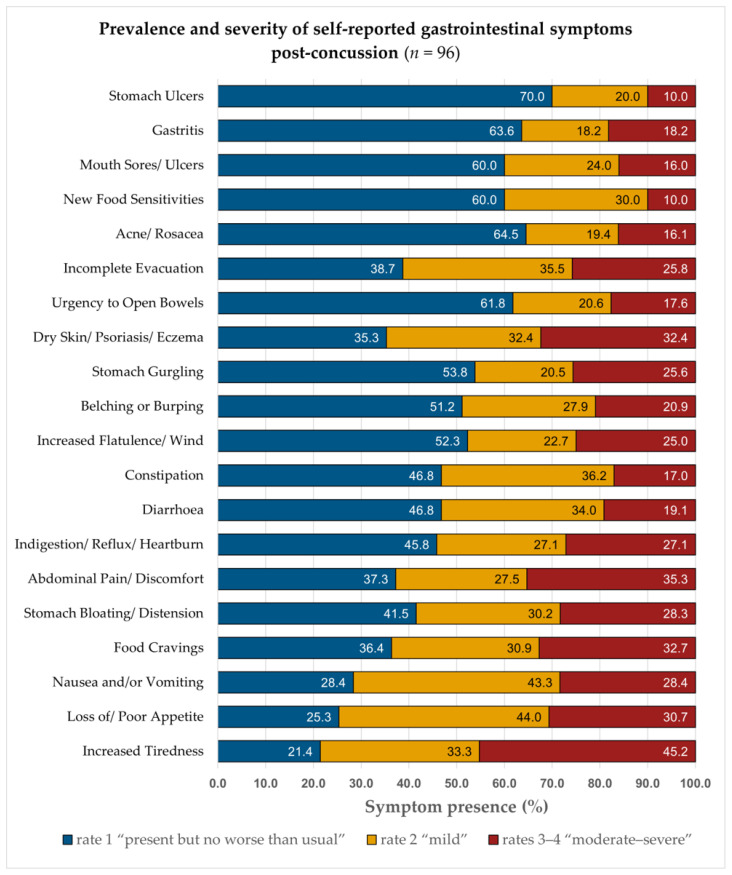
Prevalence and severity distribution of self-reported gastrointestinal (GI) symptoms post-concussion among symptomatic participants (*n* = 96).

Figure 1 shows the distribution of symptom presence and absence. Of these ratings, 880 (45.8%, orange bars) indicated some level of disturbance (ratings 1–4), with females accounting for 532 (60.5%) and males 348 (39.5%). The remaining 1040 ratings (54.2%, blue bars) reflected absence (rating = 0). Figure 2 illustrates the prevalence and severity of self-reported symptomatic ratings: 375 (19.5%) were rated 1 (present but no worse than usual), and 505 (26.3%) were rated 2–4 (mild to severe), with 234 (12.2%) rated 3–4 (moderate to severe).

The bar chart displays, for each of the 20 GI symptoms, the proportion of 1920 total GI symptom ratings provided by 96 of the 106 participants who reported ≥1 GI symptom post-SRC. Of these ratings, 880 (45.8%, orange bars) indicated some level of GI disturbance (ratings 1–4), with 532 (60.5%) reported by female participants and 348 (39.5%) by male participants. The remaining 1040 ratings (54.2%, blue bars) reflected absence of the symptom (rating = 0) following SRC; corresponding prevalence and severity distributions are described in the Results (Section 3.4.1).

Severity ratings (1–4) are displayed for each of the 20 GI symptoms, with Rating 1 (“present but no worse than usual”, blue bars), Rating 2 (“mild”, orange bars), and Ratings 3–4 (“moderate–severe”, red bars). Percentages represent the proportion of the 96 symptomatic participants endorsing each severity level for each symptom. This figure enables comparison of the frequency and severity of all GI symptoms experienced following SRC; corresponding prevalence and severity distributions are described in the Results (Section 3.4.1).

#### 3.4.1. GI Symptom Burden and Severity Post-Concussion

GI symptom burden was assessed using two thresholds: (1) any symptom presence (ratings 1–4; *n* = 96), and (2) mild-to-severe symptoms (ratings 2–4; *n* = 88). Across all severities, participants reported an average of 8.3 ± 6.2 symptoms (median 7; range 0–20), decreasing to 4.8 ± 4.7 (median 3) for rating 2–4, indicating a clinically relevant burden. Overall, 62.5% (60/96) reported at least one moderate-to-severe symptom (ratings 3–4), with an average of 2.2 ± 3.1 symptoms (median 1).

Most participants experienced a low-to-moderate burden. Among those reporting GI symptoms, 33.3% (32/96) had one–five symptoms (ratings 1–4), and 52.3% (46/88) had one–five symptoms at ratings 2–4. However, a notable subgroup reported high burden, with 44.8% (43/96) experiencing ≥10 symptoms at any severity (ratings 1–4) and 30.0% (18/60) reporting ≥5 moderate-to-severe symptoms (rating 3–4; *n* = 60). Fatigue, appetite loss, and nausea were among the most prevalent and severe GI symptoms (for frequency distributions, see Figure 2).

Symptom severity was further quantified using the Symptom Severity Index (SSI; 0–1 scale, with 1 indicating maximum severity; Table 3). The highest-ranked symptoms were increased tiredness (SSI = 0.63), incomplete evacuation (SSI = 0.56; reported by 36 (37.5%, out of 96), ranked second overall), nausea and/or vomiting (SSI = 0.54), stomach bloating/distension (SSI = 0.54), loss of/poor appetite (SSI = 0.52), and food cravings (SSI = 0.52), reflecting a fatigue and appetite-related symptom cluster. Two participants reported additional GI-related issues not captured by the survey response options, including scalp irritation with severe flaking (rating = 4) and increased vomiting post-injury (rating = 3), illustrating individual variability in systemic and GI responses post-concussion.

#### 3.4.2. Sex-Based Differences in GI Symptom Reporting

Sex-based differences were examined among 96 participants (41 males, 55 females; Table 4). Most symptoms were reported at similar rates across sexes, with effect sizes ranging from negligible to moderate (ARD 0–20%). Chi-square analyses identified two symptoms that were significantly more prevalent in females: loss of or poor appetite (*n* = 46, 83.6% vs. *n* = 25, 61.0% (*χ^2^* (1, *N* = 96) = 6.26, *p* = 0.012, *ARD* = + 22.7%), and stomach bloating/distension (*n* = 35, 63.6% vs. *n* = 16, 39.0% (χ^2^ (1, *N* = 96) = 5.71, *p* = 0.017, ARD = + 24.6%).

When restricted to moderate-to-severe symptoms (ratings 2–4), females also reported higher prevalence of stomach bloating/distension (*n* = 23, 41.8% vs. *n* = 9, 22.0%, χ^2^ = 4.17, *p* = 0.040) and abdominal pain/discomfort (*n* = 20, 36.4% vs. *n* = 7, 17.1%, χ^2^ = 4.32, *p* = 0.038.

Trends toward higher prevalence in females were observed for tiredness (*n* = 42, 76.4% vs. *n* = 24, 58.5%, ARD = +17.8%, *χ^2^* = 3.47, *p* = 0.060) and food cravings (*n* = 23, 41.8% females vs. *n* = 12, 29.3% males, ARD = +12.5%, χ^2^ = 1.60, *p* = 0.200). Chi-square analyses indicated higher prevalence in females for loss of/poor appetite (*p* = 0.012) and stomach bloating/distension (*p* = 0.017). Mann–Whitney tests showed higher severity for stomach bloating/distension in females (Z = −1.92, *p* = 0.054), and stomach gurgling in males (Z = −2.00, *p* = 0.046).

#### 3.4.3. Post-Injury GI Symptom Experience

Among participants reporting ≥1 symptom (*n* = 96), the most frequent were increased tiredness (87.5%, mean 2.19 ± 1.32), loss of/poor appetite (74.0%, mean 1.53 ± 1.18), and nausea/vomiting (74.0%, mean 1.59 ± 1.24). Food cravings (57.3%), stomach bloating/distension (53.1%), and abdominal pain (51.0%) were also common, while stomach ulcers (20.8%) and gastritis (22.9%), were less frequent and of lower severity.

When restricting to moderate-to-severe symptoms (ratings 2–4) among 88 participants (91.6%), increased tiredness remained most prevalent (68.8%, mean 2.91 ± 0.87), followed by loss of/poor appetite and nausea/vomiting (54.2%, each). Food cravings (36.5%), bloating (33.3%), and abdominal pain (28.1%) were also common at higher severities. Mean severity scores for these symptoms ranged from 2.42 to 2.91 (SD = 0.67–0.98), indicating moderate-to-severe intensity. Highlighting that GI disturbances are common post-SRC, with fatigue, appetite loss, and nausea being most prevalent and severe. Detailed descriptive data are provided in Supplementary Table S5a,b.

### 3.5. Post-Concussion Symptoms Within 24 Hours

Within the first 24 h post-concussion, 105 participants (58 females, 47 males) reported 1558 symptoms on the RPQ (rated 1–4; Table 5). Females contributed 928 symptom data points (51.9%) and males 752 (44.8%). All participants reported headaches (100.0%, mean 3.04 ± 0.85), while fatigue (95.2%, mean 2.84 ± 1.14), and poor concentration (95.2%, mean 2.57 ± 1.18) were also prevalent and moderate to severe. Females reported slightly more symptoms than males (50/58, 86.3% vs. 37/47, 79.7%). Other frequent symptoms included dizziness (91.4%, mean 2.57), irritability (87.7%, mean 2.24), and sleep disturbances (83.8%, mean 2.03). Less severe symptoms included noise sensitivity (85.7%), light sensitivity (82.9%), and frustration (81.9%), with mean severities of 2.09–2.24, while double vision was the least severe (55.2%, mean 1.01).

Significant sex differences were observed for feeling depressed/tearful (*χ^2^* (1) = 4.70, *p* = 0.030), with females reporting more frequently than males (52 vs. 35). Borderline differences were noted for irritability (*χ^2^* (1) = 3.12, *p* = 0.077), noise sensitivity (*χ*^2^ (1) = 3.95, *p* = 0.050), light sensitivity (*χ*^2^ (1) = 2.99, *p* = 0.080), restlessness (*χ*^2^ (1) = 3.01, *p* = 0.083), and double vision (*χ*^2^ (1) = 3.08, *p* = 0.080), all generally higher in females. No other symptoms differed significantly by sex. Additionally, 39 participants (30%) reported other symptoms spanning physical, cognitive, psychological, balance/coordination, and appetite domains, generally moderate to severe.

Overall, these findings indicate a high burden of post-concussion symptoms within 24 h, predominantly physical and cognitive, with sex differences observed for a subset of symptoms.

#### Nausea Recognition Across Symptom Tools (RPQ and GI Items)

Participants reported nausea following SRC at two timepoints using the RPQ (Q32) and a GI-specific symptom items (Q38). In the GI section, 71/106 participants (66.0%) reported nausea/vomiting, including 40 females (67.8%) and 31 males (66.0%). On the RPQ, 80/106 participants (75.5%) reported nausea, with similar sex proportions: 45/59 females (77.6%), 35/47 males (74.5%).

Interestingly, 22 participants (20.8%) did not report nausea on either tool (Q32 and Q38 = 0). However, there were discrepancies in nausea reporting. Thirteen participants rated nausea only on the RPQ (Q32), including six who reported moderate-to-severe ratings (≥2), while four participants reported nausea exclusively on the GI-specific item. Among the 67 participants (63.2%) reporting nausea on both tools, 10 (14.9%) rated it more severely on the GI item, 24 (35.8%) rated it higher on the RPQ, and 33 (49.3%) reported equal severity across both tools. These findings suggest that framing nausea within a GI-specific context may influence symptom recognition for a subset of participants.

### 3.6. GI Function at 3 and 6 Months Post-SRC, and at ToSC

Time since participants’ most recent concussion varied widely (median 24 months; IQR 6–60; range 0.03–216 months), resulting in different numbers contributing data at the 3- and 6-month post-concussion timepoints compared with ToSC.

At 3 months post-concussion (*n* = 88), 32 participants (36.1%) reported problematic GI function, including flatulence, incomplete evacuation, looser stools, and constipation, while 56 (63.6%) reported normal or good function. Among those with poor function, the median time since concussion was 24 months (IQR 11–72).

At 6 months post-concussion (*n* = 79), 23 participants (29.1%) reported problematic GI function, with symptoms such as bloating, reflux, flatulence, diarrhoea, appetite dysregulation, and new food sensitivities or gastritis; 56 (70.9%) reported normal or good function. Among those with poor function at 6 months, the median time since concussion was 48 months (IQR 12–72). Six participants who reported normal or good GI function at both 3 and 6 months subsequently reported poor function at ToSC; their most recent concussion ranged from 7 to 180 months prior (median 37.5 months).

At ToSC (*n* = 106), 28 participants (26.4%; 21 females, 7 males) reported poor GI function a median of 15.5 months post-concussion (IQR 7–60), with ongoing mild to moderate symptoms such as flatulence, bloating, reflux, diarrhoea, and food sensitivities. Sixteen participants described having ok, manageable, or occasional symptoms (e.g., bloating, heartburn) or were awaiting clinical consultation, while 63 participants (59.4%, 30 females, 33 males) reported normal or good function (median 24 months; IQR 6–60).

#### 3.6.1. Pre- and Post-Concussion Gastrointestinal Health

##### Food Intolerances and Sensitivities

Prior to concussion, 26 participants (23.1%; 76.9% female, *n* = 20) reported food intolerances or sensitivities: 50.0% (*n* = 13) self-reported, 46.2% (*n* = 12) professionally diagnosed, and 1 unclear blood test result. Common triggers included dairy or lactose (38.5% *n* = 10), gluten or wheat (7.7%, *n* = 2), soy (7.7%, *n* = 2), shellfish (3.8%, *n* = 1), and specific fruits or vegetables (15.4%, *n* = 4; e.g., bell peppers, oranges, pineapples, kiwi). Three participants (11.5%) reported difficulty digesting meats or protein-rich foods (e.g., pork, red meat), and 3 (11.5%) had peanut allergies. Four (15.4%) reported multiple or undetermined sensitivities and were awaiting clinical investigation.

At ToSC following concussion, 14 participants (13.5%; 64.3% female, *n* = 9) reported new or worsened intolerances or sensitivities, most self-diagnosed (78.5%, *n* = 11). Common triggers included gluten, wheat, or starch products (28.6%, *n* = 4; e.g., bread, pasta), dairy (*n* = 5), acidic foods, eggs, and bananas (7.1%, *n* = 1). Associated symptoms included bloating, irritable bowel syndrome with constipation (IBS-C; 7.1%, *n* = 1), appetite loss, stomach pain (14.2%, *n* = 2). Three (23.6%) were undergoing investigations without an identified trigger.

##### Gastrointestinal Conditions

Prior to concussion, nine participants (6.9%; 88.9% female) reported diagnosed GI conditions, including IBS (*n* = 3), food protein-induced enterocolitis syndrome (FPIES; *n* = 1), a functional GI disorder (*n* = 1), a structural GI condition (*n* = 1), and polycystic ovary syndrome (PCOS)-related GI symptoms (*n* = 1). Most participants (91.5%, *n* = 97) reported no known GI conditions.

Following concussion, 18 participants (16.9%; 72.0% female) reported new or worsened GI symptoms or conditions, including diarrhoea (*n* = 6), IBS or IBS-C (*n* = 3), inflammatory bowel disease (IBD) confirmed by colonoscopy (*n* = 1), a PCOS-related colitis flare (*n* = 1), gastroenteritis (*n* = 1), constipation related to prior gut surgery (*n* = 1), acute nausea (*n* = 1), GI sensitivity or dyspepsia (*n* = 2), and unresolved GI issues under investigation (*n* = 2). Symptom onset ranged from immediate to delayed, with some participants reporting overlapping symptoms.

##### Supplement and Functional Food Use for GI Management

Prior to concussion, 88 participants (83.2%) reported no probiotic use, 5 (4.7%) reported occasional use, and 13 (12.3%) reported regular use of dietary supplements or probiotic-rich foods (e.g., yoghurt, kefir). At ToSC, regular probiotic use increased to 22 participants (20.1%), while non-use declined to 81 (76.4%).

Use of probiotics and functional foods was lowest among participants reporting good or very good GI function (>90% reporting no use). In contrast, use was higher among those with moderately good GI function, increasing from five participants (12.2%) prior to concussion to nine (22.0%) at ToSC, and among those with poor GI function, increasing from two (10.5%) to four (21.1%). Functional food and supplement use showed similar patterns.

At ToSC, participants reported dietary changes and the use of supplements or functional foods to support digestive function and GI health, including probiotics, glutamine, digestive enzymes, magnesium, fibre supplements, high-fibre foods (e.g., whole grains), and fermented foods (e.g., kefir, yoghurt, probiotic drinks, miso, kombucha, sourdough, pickled vegetables). Some participants reported using probiotic products post-concussion to manage bloating or using magnesium for constipation.

No evidence in the data suggested that pre-concussion probiotic use was associated with reduced post-concussion GI symptoms.

Only 15 participants (13.1%) reported access to a nutrition professional, with 10 receiving support post-concussion. Most participants (*n* = 60, 80.0%) reported no support, and 31 (29.3%) did not respond, indicating limited access to nutrition care post-concussion.

## 4. Discussion

In this sample of Irish athletes with a history of SRC, GI disturbances were highly prevalent, 90.6% (*n* = 96/106) reported at least one symptom. The most frequently reported symptoms were appetite loss, nausea/vomiting, fatigue, and changes in bowel habits (increases in harder [types 1–2] and softer stools [types 5–7]). As shown in Figure 1 and Figure 2, almost half of all GI ratings indicated disturbance (45.8%), and several symptoms (e.g., appetite loss, nausea, abdominal pain, bloating) were frequently rated moderate-to-severe (3–4). Table 2 shows post-SRC variability in stool form and frequency, indicating fluctuations in bowel habits. Collectively, these descriptive findings suggest that GI complaints form a notable component of the post-concussive symptom experience for many athletes.

Sex-based differences were evident. Females reported a higher GI symptom burden for appetite loss, bloating, and abdominal pain, while nausea prevalence was similar across sexes. These patterns indicate that sex may influence GI symptom presentation and support consideration of sex-aware assessment, monitoring, and subsequent management strategies. Current concussion tools (e.g., SCAT6) include minimal GI-related items, which may contribute to under-recognition of these complaints and sex-specific profiles [1,7].

This study contributes to evidence that GI symptoms are common post-SRC and may be under-recognised in current screening practices. Mechanisms cannot be determined from these data; however, the frequency and severity patterns in Figure 1 and Figure 2 and stool-form and frequency variability in Table 2 highlight the breadth of athletes’ self-reported GI disturbances post-SRC. Including GI symptom checks and bowel-habit monitoring may help identify athletes who require follow-up or nutritional support and enable individualised recovery planning. Prospective studies are needed to establish clinical significance. Notably, approximately one-quarter of athletes reported ongoing symptoms at ToSC, and females reported a higher GI burden, suggesting potential sex-specific considerations for further research.

### 4.1. Gastrointestinal Symptom Burden

Athletes reported 298 SRC events; among the 96 symptomatic participants, 88 (91.6%) endorsed at least one mild-to-severe GI symptom. Appetite loss, nausea/vomiting, fatigue, and changes in bowel habits are the most prominent disturbances (see Figure 1 and Figure 2). Appetite-related symptoms are not routinely captured in post-injury screening tools, and may be underreported, and influence fuelling and energy availability [4,27,35].

Although most athletes reported symptom improvement over time, 26.4% described ongoing GI issues at ToSC (e.g., bloating, altered bowel habits, food sensitivities). Several athletes reported using reactive strategies (e.g., dietary changes) to mitigate their symptoms. Only 13.1% reported receiving dedicated nutrition support, suggesting limited access to nutritional input during SRC recovery. Retrospective reports highlight strategies used in practice with athletes during periods of reduced food intake, such as liquid-based meals or frequent snacks to maintain energy intake during periods of low appetite [4].

Formalising brief GI symptom checks (e.g., appetite change, nausea/vomiting, bowel changes) and considering referral pathways to nutrition support may aid follow-up and symptom management. Prospective studies are needed to determine clinical significance.

### 4.2. Disrupted Bowel Function and Stool Consistency

Athletes presented greater variability in bowel patterns post-SRC (Table 2). Stool frequency declined slightly, with significant increases in both infrequent and loose/watery bowel movements. Stool-form analysis revealed a decrease in normal stools (BSFS type 3–4), an increase in softer stools (type 5), and the emergence of watery stools (type 7). Although the decline in bowel movement frequency (3.90 ± 1.13) was statistically significant, the small absolute difference (approximately 0.3 movements/day) and modest effect size suggest limited clinical impact.

The stool-form and frequency changes observed may have practical relevance for athlete care. Softer stools can increase fluid loss, whereas harder stools may reflect reduced transit time or fluid retention [36,37]. Both patterns can influence hydration status, digestion, and nutrient availability, which may affect fuelling and tolerance to rehabilitation [4,27]. Individual differences in vagal tone, stress response, microbiota composition, autonomic function, and immune status may also modulate susceptibility to GI and neurological symptoms post-SRC [24,25]. These patterns align with proposed mechanisms involving ANS and ENS dysregulation and disrupted GBA signalling (e.g., sympathetic activation, neuroinflammation); however, these mechanisms were not measured in this study and require prospective confirmation [38,39]. Such pathways could influence bowel symptoms and emotional regulation (e.g., altered serotonin production in the gut). Physiologically, acute SRC has been associated with sympathetic activation and reduced splanchnic blood flow, which may impair gut motility and contribute to softer stools, infrequent bowel movements, or delayed gastric emptying [23,24,40]. Neuroinflammation, oxidative stress, and mitochondrial dysfunction may also further exacerbate these GI disturbances [2,20] but were not evaluated in the present study.

### 4.3. Sex-Based Differences in GI Symptoms

Female athletes experienced greater appetite loss, along with increased bloating and abdominal discomfort, highlighting the need for sex-specific assessment and recovery strategies. Hormonal fluctuations in oestrogen and progesterone may influence gut motility, visceral sensitivity, immune responses, and gut microbiome composition, which may explain observed sex differences [15,18,24,25,26,27,28]. Female athletes also reported a higher overall post-SRC symptom burden, consistent with previous research [6,41]. Notably, females athletes reported more pre-existing and post-injury dietary sensitivities. Although the underlying reasons cannot be determined from these data, prior work has suggested that factors such as ANS regulation, hormonal changes, visceral sensitivity, gut motility, and symptom expression may contribute to sex-related differences in GI experiences [24,25]. 

Beyond GI symptoms, female athletes also reported a greater overall post-SRC symptom burden, including dizziness, sleep disturbances, and cognitive impairments, consistent with established disparities in SRC outcomes [15,18,42]. Although males had a greater high-contact sport exposure (78.2% vs 61.8%), females still accounted for 49.0% of SRCs (146/298), consistent with evidence that females may experience more frequent or severe PCS [18,43,44]. Heightened visceral pain sensitivity and stress-related GI dysfunction may amplify symptom burden experienced by females, underscoring the need for tailored nutritional and clinical interventions during recovery. Future research should focus on understanding the biological and psychosocial mechanisms driving these sex differences to evaluate whether targeted strategies could support recovery outcomes for female athletes.

### 4.4. Critical Gaps in Concussion Care

Despite the high prevalence of self-reported GI symptoms in this sample, current SRC protocols (e.g., SCAT6) include limited GI content [1]. Appetite loss, bowel-habit changes, and new or worsened food intolerances may represent missed monitoring opportunities in routine follow-up and recovery strategies. Nutrition professionals appear infrequently involved, with 13.1% of athletes reporting access. Evaluating whether structured GI screening and nutrition referral pathways add clinical value should be a priority for future research. Nutrition professionals are rarely embedded within concussion-management teams [4,45], limiting athletes’ access to tailored dietary strategies; assessing the potential benefits of integrating sports performance dietitians or nutritionists into multidisciplinary SRC care warrants further investigation.

### 4.5. Clinical Implications and Future Directions

Consider integrating brief GI-symptom checks (appetite change; nausea/vomiting; stool form or bowel-habit change; food tolerance) into post-SRC follow-up to support early identification and monitoring. Where symptoms persist, referral pathways to sports performance dietitians and nutritionists may assist daily intake management and provide pragmatic strategies, such as smaller, more frequent meals; liquid-based options; and correction of deficiencies [4,45]. GI and microbiota-support strategies should be evaluated prospectively and, where appropriate, in randomised trials to establish clinical relevance.

Standard concussion frameworks give limited attention to GI disturbances and focus mainly on nausea/vomiting; consequently, issues such as bloating or altered bowel habits may be under-recognised. A small number of respondents also reported GI concerns outside the main survey items, underscoring the heterogeneity of post-SRC experiences and the potential value of individualised assessment during recovery.

Nutrition professionals, including sports performance dietitians and nutritionists, are well-positioned to support athletes with ongoing GI symptoms; however, their involvement is limited in current pathways [4,45]. Cognitive impairment and dysregulation post-SRC may further complicate routine dietary decision-making for some athletes, affecting tolerance of intake and day-to-day fuelling [4]. These observations suggest a potential care opportunity that warrants further investigation.

Future research should use longitudinal designs to track symptom evolution and include objective GI assessments (e.g., stool diaries, validated GI symptom scales) and relevant biomarkers (e.g., microbiome profiles, inflammatory markers). Studies should also systematically capture key confounders such as dietary changes, hydration, medications, stress/anxiety or other mental health diagnoses, menstrual cycle factors, and pre-existing GI conditions (e.g., IBS/IBD). Additional gut-health variables (e.g., microbiota composition, short-chain fatty acids) may be explored where appropriate. Controlled trials evaluating pragmatic nutrition and probiotic interventions, considering sport- and sex-specific patterns, are needed. Longitudinal studies should clarify whether ongoing post-SRC GI symptoms contribute to long-term GI issues. Multidisciplinary follow-up, including referral to dietetic or sports nutrition support where symptoms persist, may be feasible [4,45] and warrants prospective evaluation.

### 4.6. Limitations

This study has several limitations. First, convenience sampling may limit generalisability and introduce selection bias, as athletes with greater interest in concussion, greater exposure, or participation in high-risk sports may have been more likely to respond.

Second, the online, retrospective, self-report cross-sectional design, together with the wide time-since-injury range (0.03–216 months), prevents causal inference and introduces potential recall bias or inaccuracies, as participants reflected on both their post-concussion experiences and current GI status at ToSC. Time since the most recent concussion varied widely and was used descriptively to provide context for symptom reporting. Nevertheless, response completeness was high, with minimal missing data handled using pairwise analysis.

Third, several unmeasured confounders that may influence GI symptom reporting were not captured, including dietary intake/changes, hydration, medication use, stress/anxiety or other mental health diagnoses, menstrual cycle factors, and recent travel. The absence of these covariates introduces the risk of residual confounding.

Fourth, the study population was limited to athletes in Ireland, which may restrict applicability to other sporting and/or healthcare contexts. Cross-cultural and healthcare system differences may influence concussion management and symptom reporting, although widely adopted tools such as SCAT6 will help improve standardisation.

Survey data cannot uncover underlying biological and psychosocial mechanisms. Despite these limitations, our findings support the integration of routine GI symptom screening and nutritional management into post-SRC care. Athlete-centred, multidisciplinary follow-up, including input from nutrition professionals, should be prioritised across both elite and amateur sporting settings.

## 5. Conclusions

In this sample of Irish athletes with a history of SRC, self-reported GI disturbances were prevalent, including appetite loss, nausea, bloating, and altered bowel patterns. Female athletes reported a higher GI symptom burden. These findings are descriptive and do not establish causation, but they suggest that GI complaints may be under-recognised in current concussion pathways.

Current concussion protocols contain minimal GI-related content, and few athletes reported access to nutrition support during recovery. GI-symptom checks and facilitated referral to sports performance dietitians and nutritionists where symptoms persist may be feasible components of multidisciplinary, athlete-centred follow-up; however, prospective evaluation is needed to determine clinical relevance and impact.

Future research should incorporate objective GI measures, evaluate targeted dietary interventions, and explore sex-, sport-, and injury-specific patterns to guide evidence-based refinements to concussion care.

## 6. Practical Applications

- Integrate GI-symptom checks (appetite change; nausea/vomiting; stool form or bowel-habit change; food tolerance) within post-SRC follow-up frameworks to support early identification and monitoring.
- Where athletes experience symptoms post-SRC, referral to sports performance dietitians or nutritionists may be employed to support day-to-day intake management and pragmatic strategies (e.g., smaller, more frequent meals; liquid-based options; correction of deficiencies).
- Evaluate these approaches prospectively, including randomised trials where appropriate, to determine clinical relevance and patient-centred outcomes; if supported, targeted nutrition strategies could then be considered for integration into concussion-care pathways.

## Figures and Tables

**Table 1:** Participant characteristics by sex, sport participation, and concussion history (*N* = 106).

Variable	Total (*N* = 106)	Female	Male
Sex assigned at birth, *n* (%)	–	59 (55.7) ^a^	47 (43.4)
Age (years), mean ± SD	26.4 ± 7.6	26.6 ± 7.8	26.2 ± 7.4
Total Sports Played, n (%)	281 (100.0)	157 (55.9)	124 (44.1)
Sport Contact Level, n (%) ^b^			
High Contact	194 (69.0)	97 (61.8)	97 (78.2)
Moderate Contact	46 (16.4)	36 (22.9)	10 (8.1)
Minimal-to-Non	41 (14.6)	24 (15.3)	17 (13.7)
Sport Type, n (%) ^b^			
Stick-Based Team	36 (12.8)	25 (15.9)	11 (8.9)
Player-Based Team	161 (57.3)	79 (50.3)	82 (66.1)
Individual	14 (5.0)	7 (4.5)	7 (5.6)
Combat	45 (16.0)	28 (17.8)	17 (13.7)
Extreme	25 (8.9)	18 (11.5)	7 (5.6)
**Concussion (SRC) History**			
Last SRC time reported, *n* (%)	105 (99.1)	59 (100.0)	46 (97.9)
Last SRC time (Mo), mean ± SD ^c^	39.6 ± 44.3	31.1 ± 33.7	50.5 ± 53.5
Total SRCs reported (all, 1–9+), n (%) ^d^	298 (100.0)	146 (49.0)	152 (51.0)
SRC total, mean ± SD	2.8 ± 2.3	2.5 ± 1.8	3.2 ± 2.6
Self-diagnosed SRCs, n (%) ^e^	103 (34.6)	48 (46.6)	55 (53.4)
Self-diagnosed, mean ± SD	1.0 ± 1.6	0.8 ± 1.5	1.2 ± 1.8
Professionally diagnosed SRCs, n (%) ^e^	195 (65.4)	98 (50.3)	97 (49.7)
Professionally diagnosed, mean ± SD	1.8 ± 1.7	1.7 ± 1.3	2.1 ± 2.1

Note: Values are presented as *n* (%) unless otherwise stated. SRC = Sport-Related Concussion; SD = Standard Deviation; Mo = months. ^a^ Two participants reported a gender identity of “woman” and were categorised as female for sex-based analysis. ^b^ Participants could report multiple sports; therefore, contact-level and sport-type reflect the number of sports/activities reported (n = 281). Of these, 90 participants reported high-contact sports (194/281), 31 moderate-contact sports (46/281), and 30 minimal-to-non-contact sports (41/281). ^c^ Time since last SRC ranged from 0.03 to 216 months (median 24 months; IQR 6–60 months). Mode for last SRC was 72 months (*n* = 105). ^d^ Four participants (1 female, 3 males) reported between 10 and 13 SRCs; “9+” responses were coded as 10 for analysis. ^e^ Percentages for Self-diagnosed SRCs (48/103; 55/103) and Professionally diagnosed SRCs (98/195; 97/195) are calculated row-wise, not by column totals.

**Table 2:** Distribution of bowel movement frequency and stool-form type (Bristol Stool Scale, Types 1–7) at ToSC and post-SRC (*N* = 106).

		**Stool-Form Type (Bristol Stool Scale)**							
**Bowel Movement** **Frequency**	**Timepoint**	**Type 1**	**Type 2**	**Type 3**	**Type 4**	**Type 5**	**Type 6**	**Type 7**	**BMF** **Total *n* (%)**
		***n* (%)**	***n* (%)**	***n* (%)**	***n* (%)**	***n* (%)**	***n* (%)**	***n* (%)**	
**1×/week**	- ToSC	–	–	1 (0.9)	1 (0.9)	–	–	–	2 (1.9)
	- post-SRC	–	–	2 (1.9)	–	1 (0.9)	1 (0.9)	–	4 (3.8)
**1× every 4–6 days**	- ToSC	1 (0.9)	–	–	1 (0.9)	–	–	–	2 (1.9)
	- post-SRC	3 (2.8)	1 (0.9)	1 (0.9)	2 (1.9)	–	1 (0.9)	–	8 (7.5)
									*p* = 0.052
**1× every 2–3 days**	- ToSC	1 (0.8)	5 (4.7)	5 (4.7)	1 (0.9)	2 (1.9)	–	–	14 (13.2)
	- post-SRC	4 (3.8)	5 (4.7)	2 (1.9)	3 (2.8)	4 (3.8)	1 (0.9)	–	19 (17.9)
**1×/day**	- ToSC	–	2 (1.9)	26 (24.5)	21 (19.8)	-	1 (0.9)	–	50 (47.2)
	- post-SRC	–	4 (3.8)	18 (17.0)	14 (13.2)	6 (5.7)	2 (1.9)	1 (0.9)	45 (42.5)
**2–3×/day**	- ToSC	–	4 (3.8)	15 (14.2)	9 (8.5)	1 (0.9)	3 (2.8)	–	32 (30.2)
	- post-SRC	–	3 (2.8)	11 (10.4)	4 (3.8)	4 (3.8)	3 (2.8)	–	25 (23.6)
**4–6×/day**	- ToSC	–	–	2 (1.9)	3 (2.8)	–	1 (0.9)	–	6 (5.7)
	- post-SRC	–	1 (0.9)	–	1 (0.9)	–	–	2 (1.9)	4 (3.8)
**≥7×/day**	- ToSC	–	–	–	–	–	–	–	0 (0.0)
	- post-SRC	–	–	–	–	–	1 (0.9)	–	1 (0.9)
	**Total Distribution by Stool Form Type**								
		**Type 1**	**Type 2**	**Type 3**	**Type 4**	**Type 5**	**Type 6**	**Type 7**	**Total *n* (%)**
**Timepoints**	- ToSC	2 (1.9)	11 (10.4)	49 (46.2)	36 (34.0)	3 (2.8)	5 (4.7)	0 (0.0)	106 (100.0)
	- post-SRC	7 (6.6)	14 (13.2)	34 (32.1)	24 (22.6)	15 (14.2)	9 (8.5)	3 (2.8)	106 (100.0)
	*p*-value:	–	–	*p* = 0.027 ^b^	*p* = 0.050	*p* < 0.001 ^a^	–	*p* = 0.054 ^c^	–

Note: BMF = bowel movement frequency; ToSC = time of survey completion; post-SRC = following the most recent sport-related concussion. Stool form was rated using the Bristol Stool Form Scale (BSFS; Types 1–7 [30]). *p*-values were calculated using Fisher’s exact test; statistical significance was set at *p* < 0.05. ^a^ Type 3 was more common at ToSC than post-SRC (*p* = 0.027). ^b^ Type 5 was more frequent post-SRC than at ToSC (*p* < 0.001). ^c^ Type 7 showed a borderline increase post-SRC (*p* = 0.054).

**Table 3:** Prevalence and severity of post-concussion gastrointestinal symptoms among symptomatic participants (*n* = 96).

GI Symptom	Severity Ratings (1–4)				Total, % (*n*)	Mean ± SD	SSI	Severity Rank
	1, % (*n*)	2, % (*n*)	3, % (*n*)	4, % (*n*)				
Increased tiredness	21.4 (18)	33.3 (28)	19.0 (16)	26.2 (22)	87.5 (84)	2.19 ± 1.32	0.63	**1**
Loss of/poor appetite	26.8 (19)	46.5 (33)	19.7 (14)	7.0 (5)	74.0 (71)	1.53 ± 1.18	0.52	**6**
Nausea and/or vomiting	26.8 (19)	40.8 (29)	22.5 (16)	9.9 (7)	74.0 (71)	1.59 ± 1.24	0.54	**4**
Food cravings	36.4 (20)	30.9 (17)	20.0 (11)	12.7 (7)	57.3 (55)	1.20 ± 1.30	0.52	**5**
Stomach bloating/distension	37.3 (19)	27.5 (14)	17.6 (9)	17.6 (9)	53.1 (51)	1.15 ± 1.35	0.54	**3**
Abdominal pain/discomfort	44.9 (22)	32.7 (16)	10.2 (5)	12.2 (6)	51.0 (49)	0.97 ± 1.20	0.47	**9**
Increased flatulence/wind	47.9 (23)	20.8 (10)	16.7 (8)	14.6 (7)	50.0 (48)	0.99 ± 1.27	0.49	**8**
Diarrhoea	46.8 (22)	36.2 (17)	10.6 (5)	6.4 (3)	49.0 (47)	0.89 ± 1.12	0.44	**13**
Indigestion/reflux/heartburn	46.8 (22)	34.0 (16)	10.6 (5)	8.5 (4)	49.0 (47)	0.86 ± 1.08	0.45	**10**
Constipation	50.0 (22)	29.5 (13)	13.6 (6)	6.8 (3)	45.8 (44)	0.81 ± 1.09	0.44	**12**
Stomach gurgling	55.0 (22)	30.0 (12)	7.5 (3)	7.5 (3)	41.7 (40)	0.70 ± 1.02	0.42	**16**
Belching or burping	58.3 (21)	20.5 (8)	17.9 (7)	7.7 (3)	40.6 (39)	0.73 ± 1.09	0.45	**11**
Incomplete evacuation	33.3 (12)	30.6 (11)	16.7 (6)	19.4 (7)	37.5 (36)	0.83 ± 1.28	0.56	**2**
Dry skin/psoriasis/eczema	58.3 (21)	19.4 (7)	11.1 (4)	11.1 (4)	37.5 (36)	0.66 ± 1.06	0.44	**14**
Urgency to open bowels	35.3 (12)	32.4 (11)	23.5 (8)	8.8 (3)	35.4 (34)	0.73 ± 1.15	0.51	**7**
Acne/rosacea	64.5 (20)	19.4 (6)	3.2 (1)	12.9 (4)	32.3 (31)	0.53 ± 0.97	0.41	**17**
Mouth sores/ulcers	60.0 (18)	30.0 (9)	3.3 (1)	6.7 (2)	31.3 (30)	0.49 ± 0.87	0.39	**19**
New food sensitivities	60.0 (15)	24.0 (6)	4.0 (1)	12.0 (3)	26.0 (25)	0.44 ± 0.90	0.42	**15**
Gastritis	63.6 (14)	18.2 (4)	9.1 (2)	9.1 (2)	22.9 (22)	0.38 ± 0.84	0.41	**18**
Stomach ulcers	70.0 (14)	20.0 (4)	5.0 (1)	5.0 (1)	20.8 (20)	0.30 ± 0.70	0.36	**20**

Note: Participants rated GI symptoms on a 4-point severity scale: “1” = present but not worse than usual; “2–4” = increasing severity from mild to severe. Columns show the percentage (*n*) reporting each severity level. SSI = weighted severity index (0–1). SSI = weighted severity index (0–1), with severity rank based on SSI (1 = most severe). Values represent only participants who reported the symptom (responses of 0 excluded).

**Table 4:** Comparison of the prevalence and severity of gastrointestinal symptoms experienced (1–4, *n* = 96) by females and males.

GI Symptom	Total, % (*n* = 96)	Male, % (*n* = 41)	Female, % (*n* = 55)	χ^2^	P–*p*	ARD (%)	Mdn. Rank (M/F)	*Z*	S–*p*
Increased tiredness	87.5 (84)	82.9 (34)	90.9 (50)	1.37	0.242	+8.0	32.5 vs. 32.5	−1.40	0.163
Loss of/poor appetite	74.0 (71)	61.0 (25)	83.6 (46)	6.26	0.012 *	+22.7	36.0 vs. 36.0	−0.51	0.615
Nausea and/or vomiting	74.0 (71)	73.2 (30)	74.5 (41)	0.02	0.879	+1.4	34.0 vs. 34.0	−0.75	0.454
Food cravings	57.3 (55)	53.7 (22)	60.0 (33)	0.39	0.534	+6.3	29.0 vs. 29.0	−1.25	0.211
Stomach bloating/distension	53.1 (51)	39.0 (16)	63.6 (35)	5.71	0.017 *	+24.6	26.5 vs. 26.5	−1.91	0.362
Abdominal pain/discomfort	51.0 (49)	41.5 (17)	58.2 (32)	2.63	0.105	+16.7	11.5 vs. 30.5	−1.92	0.054 *
Increased flatulence/wind	50.0 (48)	48.8 (20)	50.9 (28)	0.04	0.837	+2.1	28.5 vs. 20.3	−0.37	0.712
Diarrhoea	49.0 (47)	41.5 (17)	54.5 (30)	1.61	0.205	+13.1	21.0 vs. 30.5	−0.20	0.839
Indigestion/reflux/heartburn	49.0 (47)	53.7 (22)	45.5 (25)	0.63	0.426	−8.2	31.0 vs. 31.0	−0.15	0.880
Constipation	45.8 (44)	39.0 (16)	50.9 (28)	1.34	0.248	+11.9	11.5 vs. 29.0	−1.03	0.301
Stomach gurgling	41.7 (40)	39.0 (16)	43.6 (24)	0.21	0.650	+4.6	29.5 vs. 12.5	−2.00	0.046 *
Belching or burping	40.6 (39)	46.3 (19)	36.4 (20)	0.97	0.325	−10.0	25.5 vs. 11.0	−0.51	0.611
Incomplete evacuation	37.5 (36)	31.7 (13)	41.8 (23)	1.02	0.311	+10.1	18.0 vs. 18.0	−0.48	0.631
Dry skin/psoriasis/eczema	37.5 (36)	39.0 (16)	36.4 (20)	0.07	0.790	−2.7	11.0 vs. 11.0	−0.43	0.668
Urgency to open bowels	35.4 (34)	34.1 (14)	36.4 (20)	0.05	0.822	+2.2	18.0 vs. 18.0	−0.07	0.942
Acne/rosacea	32.3 (31)	26.8 (11)	36.4 (20)	0.98	0.323	+9.5	10.5 vs. 10.5	−0.78	0.437
Mouth sores/ulcers	31.3 (30)	29.3 (12)	32.7 (18)	0.13	0.718	+3.5	9.5 vs. 9.5	−0.19	0.849
New food sensitivities	26.0 (25)	22.0 (9)	29.1 (16)	0.62	0.430	+7.1	8.0 vs. 8.0	−0.13	0.897
Gastritis	22.9 (22)	24.4 (10)	21.8 (12)	0.09	0.767	−2.6	7.5 vs. 7.5	−0.27	0.788
Stomach ulcers	20.8 (20)	22.0 (9)	20.0 (11)	0.05	0.816	−2.0	7.5 vs. 7.5	−0.80	0.423

Note: Data show the prevalence (P–*p*) and severity (S–*p*) of each GI symptom (*n* = 96) by sex. Prevalence indicates the percentage of participants reporting the symptom (rating 1–4). ARD = absolute risk difference (female% − male%); χ^2^ and P–*p* = chi-square test for prevalence differences. Symptom severity (ordinal 1–4) was compared using the Mann–Whitney *U* test (median/rank, *Z*, two-tailed *p*). Mdn. = Median. * Significant differences (*p* < 0.05) were observed for: loss of/poor appetite; stomach bloating/distension; abdominal pain/discomfort; stomach gurgling.

**Table 5:** Comparison of concussion symptoms reported on the RPQ by males and females (*N* = 105).

Common Concussion Symptom	Total Reported 1–4, % (*n*)	Male 1–4, % (*n* = 47)	Female 1–4, % (*n* = 58)	χ^2^	*p*
Headaches	100.0 (105)	100.0 (47)	100.0 (58)	∞	∞
Fatigue, tiring more easily ^a^	95.2 (100)	95.7 (45)	94.8 (55)	0.89	0.300
Poor concentration	95.2 (100)	93.6 (44)	96.6 (56)	1.29	0.300
Feelings of dizziness	91.4 (96)	91.5 (43)	91.4 (53)	0.84	0.400
Taking longer to think	89.5 (94)	87.2 (41)	91.4 (53)	1.28	0.300
Being irritable, easily angered	86.7 (91)	80.9 (38)	91.4 (53)	3.12	0.077 †
Noise sensitivity and easily upset by loud noise	85.7 (90)	78.7 (37)	91.4 (53)	3.95	0.050 †
Sleep disturbance	83.8 (88)	87.2 (41)	81.0 (47)	1.52	0.200
Feeling depressed or tearful	82.9 (87)	74.5 (35)	89.7 (52)	4.70	0.030 *
Light sensitivity or easily upset by bright light	82.9 (87)	76.6 (36)	87.9 (51)	2.99	0.080 †
Feeling frustrated or impatient	81.9 (86)	76.6 (36)	86.2 (50)	2.32	0.127
Poor memory, feeling forgetful	80.0 (84)	72.3 (34)	86.2 (50)	3.70	0.055 †
Nausea and/or vomiting ^a^	76.2 (80)	74.5 (35)	77.6 (45)	0.97	0.300
Blurred vision (unfocused, fuzzy)	73.3 (77)	72.3 (34)	74.1 (43)	0.88	0.300
Restlessness	73.3 (77)	66.0 (31)	79.3 (46)	3.01	0.083 †
Double vision	55.2 (58)	46.8 (22)	62.1 (36)	3.08	0.080 †

Note: A total of 1558 symptom reports were recorded (928 from females, 55.2%, and 752 from males 44.8%). Percentages indicate the proportion of each sex reporting the symptom (severity 1–4). Chi-square tests (*df* = 1) compare symptom frequencies between sexes. ^a^ marks symptoms included in both the RPQ and the GI-specific symptom tools (fatigue and nausea). * *p* < 0.05 = statistically significant differences. † *p* = 0.050–0.083 = flagged as borderline significance. ∞ = chi-square test not applicable.

## Data Availability

The original data are unavailable to protect participant confidentiality. However, the data presented in this article are included in the Supplementary Material. Further inquiries can be directed to the corresponding author.

## Supplementary Materials

The following supporting information can be downloaded at: https://www.mdpi.com/article/10.3390/nu18060914/s1, Table S1: Survey items used to assess the total number of self-reported concussions or mTBIs among participants (*N* = 106). Table S2: Survey items used to assess gastrointestinal (GI) symptomology administered to participants (*N* = 106). Table S3: Survey items used to assess general post-concussion symptoms (RPQ) administered to participants (*N* = 106). Table S4: Survey items used assess stool form using the Bristol Stool Form Scale (BSFS) to compare pre- and post-injury gastrointestinal (GI) function following concussion/mTBI in study participants (*N* = 106). Table S5: (a) Descriptive summary of post-concussion gastrointestinal (GI) symptoms prevalence (*n* = 96; ratings 1–4). (b) Descriptive summary of post-concussion gastrointestinal (GI) symptoms intensity (*n* = 88; ratings 2–4).

## Abbreviations

The following abbreviations are used in this manuscript:

SRC	Sport-related concussion
GI	Gastrointestinal
mTBI	Mild traumatic brain injury
ANS	Autonomic nervous system
SCAT 6	Sport Concussion Assessment Tool, 6th Edition
RTP	Return-to-play decisions
GBA	Gut–brain axis
CNS	Central nervous system
ENS	Enteric nervous system (ENS)
ToSC	Time of survey completion
RPQ	Rivermead Post-Concussion Symptoms Questionnaire
BSFS	Bristol Stool Form Scale
FGD	Functional Gastrointestinal Disorders Symptom Questionnaire
χ^2^	Chi-square test
ARD	Absolute risk difference
OR	Odds ratio
RR	Relative risk
SSI	Symptom Severity Index
ρ	Spearman’s rank-order correlation
IQR	Interquartile range
U	Mann–Whitney U
Z	Z score
*p*	*p*-value
SD	Standard deviation
Mo	Months
IBS (-C)	Irritable bowel syndrome (with constipation)
PCOS	Polycystic ovary syndrome
FPIES	Food protein-induced enterocolitis syndrome
CISG	Concussion in Sport Group
