# Supplementary material for: Gastrointestinal Symptoms After Sport-Related Concussion in Irish Athletes
Source: Nutrients. 2026 Mar 13;18(6):914. doi: 10.3390/nu18060914 (PMC13029405; doi:10.3390/nu18060914)
Supplement: Supplementary file 1 [file nutrients-18-00914-s001.zip › nutrients-4151291-supplementary.pdf]

Gastrointestinal Symptoms After Sport-Related Concussion in Irish Athletes

Emma Finnegan [1], Ed Daly [1], Katherine Hunzinger [2,3], and Lisa Ryan [1,*]

Supplementary Materials

Table S1. Survey items used to assess the total number of self-reported concussions or mTBIs among participants (*N* = 106).

**Section B:** Your experience with concussion/mTBI as an athlete.		
**Definition**	"A concussion or mild traumatic brain injury (mTBI) are head injuries that occur following a direct or indirect impact to the head, face, neck, or other body parts that passes an impulsive force to the brain. This force disrupts brain function, leading to the rapid onset of transient symptoms." [1]	
**Item No.**	**Survey Item**	**Response Format**
17	When did you have your last (most recent) concussion/ mTBI?	Free text
18	How did your concussion(s) occur?	Multiple choice: Sport; Recreation; Other (please state).
19	How many concussions/ mTBIs have you experienced?	Drop-down menu: 10 options (1–9, 9+), for analysis, "9+" was coded as 10.
20	How many of your concussions/ mTBIs were self-diagnosed?<br>* Not by a medical professional (doctor, physiotherapist etc.)	Same as above.
21	How many of your concussions/ mTBIs were professionally diagnosed?<br>* Diagnosed by a medical professional (doctor, physiotherapist etc.)	Same as above.

Note: This section of the survey was administered to Irish participants (*N* = 106) to capture the total number of self-reported concussions/mTBIs, including how many were medically diagnosed or self-diagnosed.

Table S2. Survey items used to assess gastrointestinal (GI) symptomology administered to participants (N = 106).

Item No.	Survey Item	Response Format
37	Please reflect on and rate how your digestion/ gut/ GI function was after your most recent concussion/mTBI event.	1 (Poor) to 5 (Very Good)
38	Please review the gastrointestinal (GI) symptoms or conditions below. Reflect on your experience after your concussion/ mTBI/ head injury impact/ diagnosis and indicate each of them occurred after your most concussion/mTBI.<br>**Symptom list** rated: 1. Loss of appetite, 2. Nausea/vomiting, 3. Diarrhoea, 4. Constipation, 5. Stomach ulcers, 6. Gastritis, 7. New food sensitivities, 8. Mouth sores/ulcers, 9. Indigestion/heartburn, 10. Incomplete evacuation, 11. Dry skin/eczema, 12. Acne/rosacea, 13. Food cravings, 14. Abdominal pain, 15. Bloating, 16. Flatulence, 17. Belching, 18. Gurgling, 19. Urgency to open bowels, 20. Increased tiredness	Likert scale:<br>0 = Not experienced; 1 = No more of a problem; 2 = Mild problem; 3 = Moderate problem; 4 = Severe problem.
39<br>40	Did you experience any other GI-type symptoms/ difficulties? Rate any additional symptoms listed in Q39.	Free text; Likert scale (above)
41	At 3 months post-concussion, did you experience any GI symptoms? Please state.	Free text
42	At 6 months post-concussion, did you experience any GI symptoms? Please state.	Free text
43	Currently, please describe your current gut function and digestion following recovery from/ most recent concussion/mTBI.	Free text
44	Before your concussion, were you taking a probiotic supplement? If yes, please specify type/brand/strain.	Yes/ No; Free text
45	Do you currently take a probiotic supplement? If yes, please specify type/brand/strain.	Yes/ No; Free text
46	Before your concussion were you using any dietary supplements/functional foods for GI health? If yes, please list the supplements or foods (e.g., prebiotics, glutamine, fermented foods).	Yes/ No; Free text
47	Do you currently use any supplements/functional foods for GI health? If yes, please list the supplements or foods.	Yes/ No; Free text
48	Do you have access to a performance dietitian or nutritionist, and were they informed of your concussion? If yes, how soon after?	Yes/ No; Free text

Note: This section of the survey was administered to participants (N = 106) to assess post-concussion gastrointestinal (GI) symptom prevalence and severity. Symptom items were rated using Likert scales. Open-ended questions captured additional details on symptom duration, supplement use, and access to nutrition support. The 20 GI-specific items were adapted from validated instruments: the Functional Gastrointestinal Disorders (FGD) Symptom Questionnaire and the Gastrointestinal Symptom Rating Scale (GSRS) [33]. For this study "acute phase" refers to 24–72 hours and up to 7 days after the most recent concussion/mTBI event [7].

Table S3. Survey items used to assess general post-concussion symptoms (RPQ) administered to participants (N = 106).

Item No.	Survey Item	Response Format
32	Rivermead Post-Concussion Symptoms Questionnaire (RPQ). Adapted from King et al. (1995) [30].<br>Did you experience any of the following symptoms or conditions within the initial 24 hours following your concussion/ mTBI/ head injury impact and/or diagnosis?	1 (Poor) to 5 (Very Good)
38	Please review the gastrointestinal (GI) symptoms or conditions below. Reflect on your experience after your concussion/ mTBI/ head injury impact/ diagnosis and indicate each of them occurred after your most concussion/mTBI.<br>**Symptom list** rated:<br>21. Headaches<br>22. Feelings of dizziness<br>23. Nausea and/or vomiting<br>24. Noise sensitivity, easily upset by loud noise<br>25. Sleep disturbance<br>26. Fatigue, tiring more easily<br>27. Being irritable, easily angered<br>28. Feeling depressed or tearful<br>29. Feeling frustrated or impatient<br>30. Poor memory, feeling forgetful<br>31. Poor concentration<br>32. Taking longer to think<br>33. Blurred vision (unfocused, fuzzy)<br>34. Light sensitivity or easily upset by bright light<br>35. Double vision<br>36. Restlessness	Likert scale:<br>0 = Not experienced; 1 = No more of a problem; 2 = Mild problem; 3 = Moderate problem; 4 = Severe problem.
33, 35	Did you experience any other difficulties? Rate symptoms listed (Q33 and Q35)	Free text; Likert scale (above)

Note: This section of the survey was administered to participants (N = 106) to assess post-concussion symptom prevalence and severity. Symptom items were rated using Likert scales. The Rivermead Post-Concussion Symptoms Questionnaire (RPQ) [29] was used to assessed initial post-concussion/mTBI symptom severity, including emotional, cognitive, and neurological symptoms. [30]. For this study “acute phase” refers to 24–72 hours and up to 7 days after the most recent concussion/mTBI event [7].

Table S4. Survey items used assess stool form using the Bristol Stool Form Scale (BSFS) to compare pre- and post-injury gastrointestinal (GI) function following concussion/mTBI in study participants (*N* = 106).

Section C: Your gastrointestinal (GI) function as an athlete

* Please take note that some questions may ask you to reflect on both before and after your reported recent concussion/mTBI.

Item No.	Survey Item	Timepoint	Response Format
22	Presently, how often do you pass a bowel movement? (Please select one option)	Baseline (ToSC)	**Options**: 7-point frequency scale:<br>- Once a week<br>- Once every 4–6 days<br>- Once every 2–3 days<br>- Once a day<br>- 2–3 times a day<br>- 4–6 times a day<br>- 7 or more times a day
29	During the acute phase after your concussion/mTBI, how often were you having bowel movements? (Please select one option)	Retrospective, acute phase (24–72 h to 7 days)	
23	Using the Bristol Stool Form Scale (BSFS), please indicate how your stool currently presents.	Baseline (ToSC)	**Options**: Frequency scale: BSFS Types 1–7 (see below)
30	Using the BSFS, please indicate how your stool presented during the acute phase after your concussion/mTBI.	Retrospective, acute phase (24–72 h to 7 days)	
23, 30	Bristol Stool Form Scale (adapted from Blake et al. [28]):<br>- **Type 1:** Separate hard lumps, like nuts (hard to pass)<br>- **Type 2:** Sausage-shaped but lumpy<br>- **Type 3:** Like a sausage but with cracks on the surface<br>- **Type 4:** Like a sausage or a snake, smooth and soft<br>- **Type 5:** Soft blobs with clear-cut edges (passed easily)<br>- **Type 6:** Fluffy pieces with ragged edges, a mushy stool<br>- **Type 7:** Watery, no solid pieces, entirely liquid		

Note: This section of the survey was administered to participants (*N* = 106) to assess changes in gastrointestinal (GI) function from baseline (ToSC) to post-injury (immediately following the most recent SRC/mTBI). Stool consistency was categorised using the Bristol Stool Form Scale (BSFS) [31]. For this study, "acute phase" refers to 24–72 hours and up to 7 days after the most recent concussion/mTBI event [7].

Table S5a. Descriptive summary of post-concussion gastrointestinal (GI) symptom prevalence (*n* = 96; ratings 1-4).

Rank	GI symptom	Ratings 1-4, % (*n* = 96)	Mean Severity ± SD
1	Increased Tiredness	87.5 (84)	2.19 ± 1.32
2	Loss of/ Poor Appetite	74.0 (71)	1.53 ± 1.18
3	Nausea and/or Vomiting	74.0 (71)	1.59 ± 1.24
4	Food Cravings	57.3 (55)	1.20 ± 130
5	Stomach Bloating/ Distension	53.1 (51)	1.15 ± 1.35
6	Abdominal Pain/ Discomfort	51.0 (49)	0.97 ± 1.20
7	Increased Flatulence/ Wind	50.0 (48)	0.99 ± 1.27
8	Diarrhoea	49.0 (47)	0.89 ± 1.12
9	Indigestion/ Reflux/ Heartburn	49.0 (47)	0.86 ± 1.08
10	Constipation	45.8 (44)	0.81 ± 1.09
11	Stomach Gurgling	41.7 (40)	0.70 ± 1.02
12	Belching or Burping	40.6 (39)	0.73 ± 1.09
13	Incomplete Evacuation	37.5 (36)	0.83 ± 1.28
14	Dry Skin/ Psoriasis/ Eczema	37.5 (36)	0.66 ± 1.06
15	Urgency to Open Bowels	35.4 (34)	0.73 ± 1.15
16	Acne/ Rosacea	32.3 (31)	0.53 ± 0.97
17	Mouth Sores/ Ulcers	31.3 (30)	0.49 ± 0.87
18	New Food Sensitivities	26.0 (25)	0.44 ± 0.90
19	Gastritis	22.9 (22)	0.38 ± 0.84
20	Stomach Ulcers	20.8 (20)	0.30 ± 0.70

Note: Data include 96 participants who reported ≥1 gastrointestinal (GI) symptom (10 participants who rated "0" across all 20 symptoms were excluded). Means include "0" ratings for symptoms not experienced. SD = standard deviation.

Table 5b. Descriptive summary of post-concussion gastrointestinal (GI) symptoms intensity (*n* = 88; ratings 2–4).

Rank	GI symptom	Ratings 1-4, % (*n* = 96)	Mean Severity ± SD
1	Increased Tiredness	68.8 (66)	2.91 ± 0.87
2	Loss of/ Poor Appetite	54.2 (52)	2.46 ± 0.67
3	Nausea and/or Vomiting	54.2 (52)	2.58 ± 0.72
4	Food Cravings	36.5 (35)	2.71 ± 0.79
5	Stomach Bloating/ Distension	33.3 (32)	2.84 ± 0.85
6	Abdominal Pain/ Discomfort	28.1 (27)	2.63 ± 0.84
7	Increased Flatulence/ Wind	26.0 (25)	2.88 ± 0.83
8	Diarrhoea	26.0 (25)	2.52 ± 0.77
9	Indigestion/ Reflux/ Heartburn	26.0 (25)	2.44 ± 0.71
10	Constipation	22.9 (22)	2.55 ± 0.74
11	Stomach Gurgling	18.8 (18)	2.50 ± 0.79
12	Belching or Burping	18.8 (18)	2.72 ± 0.75
13	Incomplete Evacuation	25.0 (24)	2.83 ± 0.87
14	Dry Skin/ Psoriasis/ Eczema	15.6 (15)	2.80 ± 0.86
15	Urgency to Open Bowels	22.9 (22)	2.64 ± 0.73
16	Acne/ Rosacea	11.5 (11)	2.82 ± 0.98
17	Mouth Sores/ Ulcers	12.5 (12)	2.42 ± 0.79
18	New Food Sensitivities	10.4 (10)	2.70 ± 0.95
19	Gastritis	8.3 (8)	2.75 ± 0.89
20	Stomach Ulcers	6.3 (6)	2.50 ± 0.84

Note: Data from 88 participants who reported ≥1 gastrointestinal (GI) symptoms from 2 to 4 on the severity scale (i.e., reported experiencing the symptom); responses of 0 and 1 were excluded. SD = standard deviation.
